# Safety Identifying of Integral Abutment Bridges under Seismic and Thermal Loads

**DOI:** 10.1155/2014/757608

**Published:** 2014-10-27

**Authors:** Narges Easazadeh Far, Majid Barghian

**Affiliations:** Department of Structural Engineering, Faculty of Civil Engineering, University of Tabriz, Tabriz 5166616471, Iran

## Abstract

Integral abutment bridges (IABs) have many advantages over conventional bridges in terms of strength and maintenance cost. Due to the integrity of these structures uniform thermal and seismic loads are known important ones on the structure performance. Although all bridge design codes consider temperature and earthquake loads separately in their load combinations for conventional bridges, the thermal load is an “always on” load and, during the occurrence of an earthquake, these two important loads act on bridge simultaneously. Evaluating the safety level of IABs under combination of these loads becomes important. In this paper, the safety of IABs—designed by AASHTO LRFD bridge design code—under combination of thermal and seismic loads is studied. To fulfill this aim, first the target reliability indexes under seismic load have been calculated. Then, these analyses for the same bridge under combination of thermal and seismic loads have been repeated and the obtained reliability indexes are compared with target indexes. It is shown that, for an IAB designed by AASHTO LRFD, the indexes have been reduced under combined effects. So, the target level of safety during its design life is not provided and the code's load combination should be changed.

## 1. Introduction

Integral abutment bridges (IABs) are continuous single or multispans bridges in which the jointless superstructure is connected rigidly to the abutment. So, in the longitudinal direction these two parts can be assumed as a single integral component. The rigid connection between them leads most of displacements and loads transfer from superstructure to substructure consisting of abutments and piled foundations. By eliminating the expansion joints in IABs, most problems associated with expansion joints and bearings are reduced, such as high maintenance costs, deterioration due to deciding chemicals, and impact loads. These advantages of IABs have caused them to be used throughout the world increasingly, especially, in USA, Canada, UK, and Republic of Korea. These bridges similar to conventional bridges are subjected to primary (loads live loads, dead loads, seismic loads, etc.) and secondary effects (shrinkage, creep, passive pressure, uniform temperature changes, thermal gradients, etc.). Among these loads—due to the integrity of these bridges and the resultant complexity of soil/structure interactions—uniform thermal and longitudinal seismic loads become important and have deterministic role in the behavior of IABs.

Several researchers studied the behavior of these bridges under thermal and seismic loads separately and showed the importance of these loads on the IAB's response. Tsang and England [1] investigated the soil/structure interaction of integral bridge with full height abutments. Dicleli and Albhaisi [2] studied the effect of cyclic thermal loading on the behavior of steel H-piles foundation of integral bridges. In this research the abutments of bridges are stub. Kim and Laman [3] investigated the integral abutment bridge response under the thermal loading. In the other research they studied the long-term behavior of integral abutment bridges by using numerical analysis. Tegos et al. [4] proposed two different abutment configurations to improve seismic behavior of integral bridges. Itani and Pekcan [5] and Frosch et al. [6] investigated the seismic behavior of IABs and developed design recommendations. Maleki and Mahjoubi [7] introduced a 2D finite element model for seismic analysis of retaining walls and integral bridge abutments. They also proposed a new seismic soil pressure distribution to replace the Mononobe-Okabe [8] equations. Kim [9] proposed new load combinations for the load and resistance factor design (LRFD) to design typical IABs, by developing the nominal IAB response prediction models and establishing IAB's response statistics using Monte Carlo simulation. These developed load combinations were established using reliability analysis and included dead load, live load, and thermal load due to temperature variation, temperature gradient, backfill pressure, and time-dependent effects.

As (1) Eurocode 8 [10], American Association of State Highway and Transportation Officials (AASHTO LRFD 2012) [11] and other developed bridge design codes consider temperature and earthquake loads separately in their specified load combinations for the design of bridges; (2) by knowing that the these load combinations have been primarily defined for conventional bridges with expansion joints while due to the elimination of expansion joints and due to the integration between substructure and superstructure of IABs overall performance of bridges is different from conventional bridges; (3) according to the fact that there is a temperature difference between temperature at any time of bridge life and bridge construction time temperature, hence, a uniform temperature load is applied to the bridge. Therefore, during earthquake the existence of a uniform temperature load is an obvious matter; and (4) both the uniform temperature load and earthquake load are important for integral bridges due to their structural nature and the role of the mentioned loads on these bridges. Therefore, the importance of evaluating the safety level IABs—designed by AASHTO LRFD [11] bridge designed code—under combination of temperature and earthquake loads during the design life of IABs is perceived.

For this reason, in this paper, by using structural reliability analysis conducted on a case study bridge—designed in accordance with existing AASHTO LRFD [11] code—the safety level of bridge under combination of seismic and thermal loads was evaluated during its 75-year design life [11]. Comparing the obtained results with the target safety level, it will be clear whether the target safety level for designed integral bridge—during design life (according to AASHTO LRFD)—under combined effect of seismic and thermal loads is guaranteed or not. On the other hand, the current code has been written for conventional bridges but it is used for IABs. Codes are gradually developed to consider new bridges such as IABs. If the target safety level for these bridges is not satisfied then the modification of the code's load combination is needed.

To achieve this objective, this paper reviews the basic reliability concepts and available analytical models for studying the reliability of structures under the combination of loads. These models are (1) Turkstra's rule [19]; (2) the Ferry-Borges (or Ferry Borges-Castanheta) model [20]; (3) Wen's load coincidence method [21]; and (4) scenario sampling model [22, 23].

As the reliability index is usually used to evaluate the safety level of a structure, an IAB designed based on AASHTO LRFD bridge design specification [11] was analyzed to evaluate reliability index values for its 75-year design life (a 75-year design life is used for an IAB [11]) for different limit states. As moments and shears at the interface of pile with the abutment are most affected by the temperature change that accompanies an earthquake, the bending moment and shearing limit states of piled foundation are considered in this paper. First, the reliability indexes are calculated for bridges under the effect of only earthquake load to evaluate the target reliability indexes [24]. Then by repeating these analyses for the same bridges subjected to the combination of seismic and thermal loads and comparing evaluated indexes with the target reliability indexes, whether the target safety level for designed integral bridge—according to AASHTO LRFD code under uniform temperature and earthquake loads combination—is satisfied or not will be investigated.

As mentioned above, AASHTO LRFD [11] considers earthquake load separately in the extreme event I load combination as addressed in

(1) $$\begin{array}{c} \text{Extreme}\;\text{Event}\;I=\gamma_{\text{DL}}\text{DL}+\gamma_{P_{a}}P_{a}+\gamma_{\Delta P_{a}}\Delta P_{ae}+\gamma_{\text{EQ}}\text{EQ}, \end{array}$$

where EQ, DL, *P*_*a*_, and Δ*P*_*ae*_ are seismic load, dead load, static earth pressure, and seismic earth pressure, respectively, and *γ*_DL_ = 1.25, *γ*_*P*_*a*__ = 1.5, *γ*_Δ*P*_*a*__ = 1, *γ*_EQ_ = 1 are the considered load factors.

The statistical data required for this reliability analysis are assembled from reliability literature, Ghosn et al. [24], United States Geological survey (USGS) website [16], weather website [18, 25], and Kim [9].

## 2. Basic Concepts of Structural Reliability Theory

The purpose of the structural reliability theory is, including the uncertainties associated with the member capacity and the occurrence, intensities and effect of loads that the members are subjected to during their design life. Thus, all variables contributing in the member resistance and the load effects should be represented by random variables. The minimum characteristics to define a random variable, *R*, are probability distribution function, PDF, the Mean value, $\overset{-}{R}$, and the standard deviation, *σ*_*R*_. As shown in (2), the coefficient of variation (COV) is defined as the ratio of standard deviation, *σ*_*R*_, to mean value, $\overset{-}{R}$, and bias factor, *b*_*r*_, as the ratio of the mean value, $\overset{-}{R}$, to the nominal or design value, *R*_*n*_. Consider

(2) $$\begin{array}{c} \text{COV}=\frac{\sigma_{R}}{\overset{-}{R}},\;\;b_{r}=\frac{\overset{-}{R}}{R_{n}}. \end{array}$$

Based on structural reliability theory, the safety of a structure can be achieved just when the structural resistance (*R*) exceeds the load effects (*S*). So, the reliability, *R*_*e*_, of a structure is the probability of this exceedance as follows:

(3) Re=Pr⁡[R>S],Re=Pr⁡[Z=R−S>0],or  Re=Pr⁡[Z(X1,X2,…,Xn)>0],

where *Z* is the limit state function that relates the resistance (*R*) to the load effects (*S*) for evaluating the safety level of the structure. *X*_1_, *X*_2_,…, *X*_*n*_ are the random variables associated with the resistance and the applied loads. In contrast, probability of failure, *P*_*f*_, is the probability that the safety margin, *Z*, is less than zero as follows:

(4) $$\begin{array}{c} P_{f}=\mathrm{Pr}\left[R<S\right]=\mathrm{Pr}\left[Z<0\right]=1-R_{e}. \end{array}$$

The reliability index, *β*, is usually used to evaluate the safety level of a structure. This index is related to the probability of failure as follows:

(5) $$\begin{array}{c} \beta =-\Phi^{-1}\left(P_{f}\right), \end{array}$$

where Φ is the cumulative standard normal distribution function. A general equation for the probability of failure is defined as follows:

(6) $$\begin{array}{c} P_{f}=\overset{}{\underset{Z(\{X\})<0}{\int }}f_{\left\{X\right\}}\left(\left\{x\right\}\right)dx_{1}dx_{2}\cdots dx_{n}, \end{array}$$

where {**X**} = {*X*_1_, *X*_2_,…, *X*_*n*_} is a random variables vector, *f*_{**X**}_({**X**}) is the probability density of vector {**X**}, and *Z*({**X**}) is the limit state function. As *f*_**X***i*_ is generally unknown, the evaluation of the probability of failure, *P*_*f*_, using (6) is very difficult. Therefore, based on the type of distribution function corresponding to the structural resistance (*R*) and load effects (*S*) in the limit state function (*Z*) in (3), there are several methods to evaluate the reliability index. The methods include the first order reliability method (FORM), the second order reliability method (SORM), and Monte Carlo simulation method. In this paper, the Monte Carlo simulation method was used to evaluate the probability of failure. Then by using (5), the reliability index was obtained. The Monte Carlo method creates large number simulated outcomes of a limit state. Next, by counting the number of failure events (*Z* < 0) and dividing them into the total number of simulated events, the probability of failure, *P*_*f*_, can be estimated. In this method, during each simulation, all involved variables in the limit state function are chosen (or generated) randomly [26].

The reliability index has been used to express structural risk. For this index the range of 2 to 4 is usually specified to failure of a single component for different structural application [24].

To calculate the reliability index, at first, the statistical data for all the random variables associated with the limit state function *Z* of (3) should be obtained. These data include all the uncertainties in estimating the member resistances and the load effects. According to Nowak [13] and Ellingwood et al. [15] approach a bridge member resistance capacity by a variable *R* can be defined as follows:

(7) $$\begin{array}{c} R=MFPR_{n}, \end{array}$$

where *M* is material factor representing properties such as strength and modulus of elasticity; *F* is fabrication factor including geometry, dimensions, and section modulus; *P* is analysis factor such as approximate models for estimating member capacity, idealized stress, and strain distribution models; and *R*_*n*_ is predicted member capacity using code-specified methods. Equation (7) can be used to find the mean value of *R* using (2) if the total resistance bias, *b*_*r*_, is set to be equal to the product of the mean values of *M*, *F*, and *P*. The resistance model of (7) does not directly account for member deterioration or other changes with time. Thus, all the variables are time-independent random variables.

For a bridge member (or structural system) to be safe, the resistance should be large enough for the maximum load effect that could occur within the structure's service life. Estimating the effects of the maximum loads involves a number of random variables, which may often be associated with large levels of modeling uncertainties. In particular, the intensities of the maximum loads are time-dependent random variables in the sense that longer service lives imply higher chances that the structure will be subjected to a given extreme load level. On the other hand, the projection of limited load intensity data, collected from previous measurements over short periods of time, to future return periods is associated with various levels of statistical modeling uncertainties. In addition, modeling the structure's response to the applied loads and estimating the variables that control the effects of the loads on the structure are associated with high levels of uncertainty that are independent of the return period. These modeling uncertainties are often represented by time-independent random variables. Thus, the effect of a particular load type, *i*, on a structural member may be represented as follows:

(8) $$\begin{array}{c} S_{i}=\lambda_{i}f_{i}\left(\lambda_{Qi}C_{ij}Q_{i}\right), \end{array}$$

where *S*_*i*_ is the load effect for load type *i*; *λ*_*i*_ is the analysis modeling factor that accounts for differences between measured load effects and predicted load effects; *f*_*i*_() is the analysis prediction model that converts load intensities into load effects; *Q*_*i*_ is the projected intensity variable of load type *i* for the return period of interest; *λ*_*Q*_*i*__ is the statistical modeling variable that accounts for the limitations in predicting the value of *Q*_*i*_; and *C*_*ji*_ is the analysis variables such as bridge material and geometrical properties required for executing the analysis for load type *i*. All the variables in (8) may be considered random where *Q*_*i*_ is a time-dependent random variable and the remaining variables are time-invariant. The probability density of the load intensity, *Q*_*i*_, for a given return period, *t*, can be calculated by studying the probability that *Q*_*i*_ will exceed a given value within *t*. Assuming that the occurrence of load events follows a Poisson model, the probability that the load intensity will exceed a value *x*, within a period, *t*, is represented by (1 − *F*_*Q*_*i*,*t*__[*x*]), which may be approximated as

(9) $$\begin{array}{c} \mathrm{Pr}\left(Q_{i}>x;T<t\right)=1-F_{Q_{i,t}}\left(x\right)=1-e^{\left(-tp\right)}, \end{array}$$

where *p* is the rate of exceedance per unite time. *P* is equal to the probability of exceeding *x* when *t* equals 1.0:

(10) $$\begin{array}{c} p=\mathrm{Pr}\left(Q_{i}>x\right)=1-F_{Q_{i}}\left(x\right). \end{array}$$

For extreme values of *x*, when the values of *F*_*Q*_*i*__(*x*) are close to 1.0 and *p* is calculated for one unit of time while the return period, *t*, consists of *m* units of time, (9) can be approximated as

(11) Pr⁡(Qi>x;T<t)=1−FQi,t(x)=1−e(−tp)≈1−(1−p)m=1−(FQi(x))m.

Equation (10) can be written as follows:

(12) $$\begin{array}{c} \mathrm{Pr}\left(Q_{i}<x;T<t\right)=F_{Q_{i,t}}\left(x\right)\approx {\left(F_{Q_{i}}\left(x\right)\right)}^{m}. \end{array}$$

Equation (12) indicates that the cumulative probability function for a return period of time, *t*, may be approximated by raising the cumulative probability function of the basic time period to the power, *m*.

## 3. Reliability Methods for Combination of Loads 

In general, by considering the variability of the load magnitude with time, the loads can be classified as permanent loads that are time independent and transient loads that vary with time. The minimum required characteristics to represent a time-dependent load are the rate of occurrence in time, the time duration, and the intensity of load. This kind of load can be modeled by a random process. As described before, in order to evaluate the probability of failure of the structure under the combination of load effects, the extreme value of the combined load effects is required. For this purpose, the extreme value of the combined load effects corresponding to considered limit state should be calculated. During the design life time of a structure (*T*), different individually acting time-dependent loads may be modeled as the sum of the load effect processes *X*_*i*_(*t*) and the extreme value *X*_max⁡_(*T*) is calculated as follows:

(13) $$\begin{array}{c} X_{\max}\left(T\right)=\underset{T}{\max}\left\{X_{1}\left(t\right)+X_{2}\left(t\right)+\cdots +X_{n}^{}\left(t\right)\right\}. \end{array}$$

As generally it is very difficult to obtain an exact solution of (13), some approximate analytical models exit to estimate this solution. These are (1) Turkstra's rule [19]; (2) the Ferry-Borges (or Ferry Borges-Castanheta) model [20]; (3) Wen's load coincidence method [21]; and (4) scenario sampling model [22, 23]. As in this paper the scenario sampling model is used, this model is described briefly below.

### 3.1. Scenario Sampling Method

Scenario Sampling [22, 23] method can be used for time-dependent loads combination. Compared with other methods, this method has high accuracy and can be used for any linear and nonlinear loads combinations. This method is presented based on Monte Carlo sampling.

To calculate the probability of structural failure under the combination of several time-dependent loads for a limit state function using scenario sampling method it is done as follows: with the knowledge of the rate of occurrence, time duration, and intensity of each time-dependent load and by using Poisson's distribution function to estimate the occurrence time of any event of load, first, during structure's *T*-years design life, a scenario of load occurrence is generated. Then, whenever the intensity of any load changes, the amount of limit state function is calculated. Among the limit state function amount for each T-year life, the minimum limit state function amount is selected which is related to the maximum loads effect in the structure life. This procedure is repeated *n* times. According to Monte Carlo's method, the failure probability is calculated by dividing the total states—in which their limit state function minimum amount is negative (indicating member failure)—into the total simulating cycles, *n*, and finally the reliability index is calculated from (5). The total of simulating cycles, *n*, is chosen in a way that coefficient of variation related to failure probability is maximum 2%.

## 4. IAB Pile Resistance Capacity

As described before, the considered limit state in this study is the bending moment and shearing failure of the IAB pile. For supporting the abutments of the considered IAB a single row of steel H-piles is used. As the bending moment and shearing capacities of steel pile are defined by its yield stress, *F*_*y*_, and geometric parameters, by considering the yield stress of pile section as a random variable the uncertainties associated with the bending moment and shearing capacities of the pile are taken into account during reliability analysis. So, for the yield stress, *F*_*y*_, a bias of 1.05 and a COV of 10% using a lognormal distribution are used [27].

## 5. Reliability Models for Loads

According to extreme event I load combination of AASHTO LRFD [11], nominal design loads dead load, earth pressure, seismic load, and uniform thermal load are just considered in this study. The required statistical models of these loads are described in this section.

### 5.1. Dead Load

Based on Nowak [13], the statistics for dead load are summarized in Table 1.

Since, in this study, the deck of considered IAB is a factory-made steel girder composite and cast-in place concrete slab, a bias of 1.08 = 1.03 × 1.05 with a COV of 12.8% = $\sqrt{10^{2}+8^{2}}$ using a normal distribution is used for dead load.

### 5.2. Backfill Earth Pressure

Backfill static earth pressure dependent on the movement direction of IAB's abutments under applied loads can be the form of passive or active. The backfill passive pressure (abutment moves far from backfill) resists bridge against applied load while active pressure (abutment moves toward backfill) is a permanent load on the abutments. Based on Rankin's theory, the lateral earth pressure considering cohesionless backfill soil is determined by unit weight and fraction angle of soil. Based on Becker's researches [14], statistics for backfill soil are summarized in Table 2.

### 5.3. Earthquake Load

The reliability analysis of a structural member under earthquake load involves a number of random variables that the uncertainty of them should be considered. A brief discussion of each variable is given and statistics for each are summarized in Table 3.

#### 5.3.1. Intensity of Earthquake Acceleration

**Figure 1:**
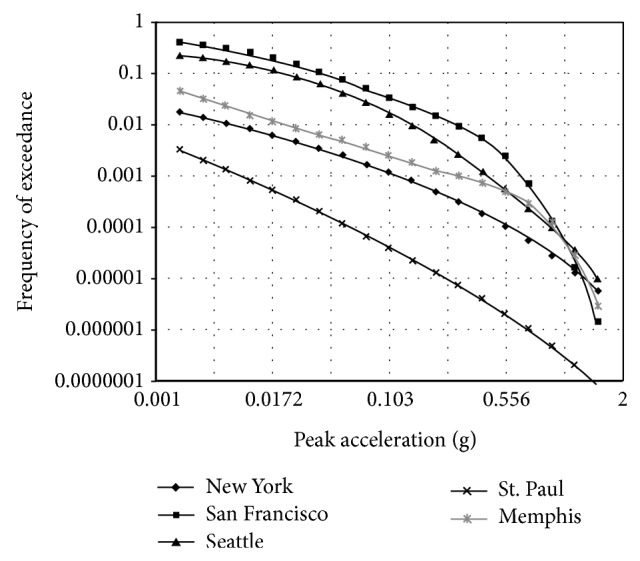
Annual probability of exceedance curves for PGA [12].

The expected earthquake intensity for IAB's sites was obtained from the USGS [16]. These maps provide the horizontal peak ground accelerations (PGAs) for various sites throughout the United States with 7 percent exceedance probability in 75 years (a return period of about 1000 yr). In this study five sites were considered as the IAB's site: San Francisco with 94117 zip code, Seattle with 98195 zip code, Memphis with 38101 zip code, New York with 10031 zip code, and St. Paul with 55418 zip code. An annual exceedance probability curves for PGA was provided by Frankel et al. [12] for a number of sites (see Figure 1).

#### 5.3.2. Rate of Earthquake Occurrence

The number of expected earthquakes varies from site to site and is available at the USGS [16]. The average number of earthquakes in one year is about 8 for San Francisco, 2 for Seattle, 0.5 for Memphis (one every 2 years), 0.4 for New York (one every 2.5 years), and 9 × 10^−3^ for St. Paul (one every 111 years).

#### 5.3.3. Natural Period of IABs

The natural period of an IAB is related to the type of bridge structure, type of bridge foundation, the characteristics of the used materials, the characteristics of bridge geometry, the interaction between soil and structure (SSI), and so forth. As the considered IAB in this study included the effects of SSI, based on published researches [17], a bias of 0.9 and a COV of 20% using a normal distribution was used for the natural period of the IAB.

#### 5.3.4. Mass Applied

To account for uncertainties associated with the mass applied on the IAB's members (considering weight alone) a bias of 1.05 and a COV of 5% using a normal distribution were used [13, 15].

#### 5.3.5. Seismic Response Coefficient

The design response spectrum proposed by AASHTO LRFD [11] was used in this paper. These design spectra are based on the USGS [16]. For considering uncertainties associated with these spectra, the statistics provided by Frankel et al. [12] were used. They found that, for all sites inside USA, the mean value of spectral accelerations is very close to the design spectral accelerations, so a bias of 1.0 can be used. Also, for all sites, COV depends on the number of observed earthquakes at which, the COV is low for sites with high frequency of earthquakes and for sites with low frequency the COV is high. Therefore, for San Francisco, the COV is about 15%, for Seattle and Memphis it is about 25%, for New York it is about 30%, and for St. Paul it is about 40%. For this variable a normal distribution is used.

#### 5.3.6. Modeling Factor

Modeling factor was used to take into account for the uncertainties produced during the dynamic analysis process. A bias of 1 and a COV of 20% using a normal distribution were used for this variable [15].

#### 5.3.7. Reliability Equation for Earthquake Load

Using the presented information, the equivalent seismic load applied on the IAB is defined as follows:

(14) $$\begin{array}{c} F_{\text{EQ}}=\lambda_{\text{eq}}C^{\prime }S_{a}\left(t^{\prime }T\right)\times \frac{A\times W}{R_{m}}, \end{array}$$

where *F*_EQ_ is the equivalent applied load, *λ*_eq_ is the modeling factor, *C*′ is the response spectrum modeling parameter, *A* is the maximum 75-year peak ground acceleration at the site, *S*_*a*_  is the calculated spectral acceleration using the IAB's period, *T*, and period modeling factor, *t*′, *W* is the weight of system, and *R*_*m*_ is the response modification factor which is equal to 1.0 for IAB's pile [11]. The statistics for random variables used in (14) are summarized in Table 3.

### 5.4. Uniform Thermal Load

Due to eliminating expansion joints in IAB's superstructure and due to the rigid connection of IAB's superstructure to substructure (consisting of abutments, pile, and backfill soil), the movements of IAB's superstructure due to temperature variation are transferred to substructure and induce thermal load on substructure. This uniform thermal load depends on the superstructure's temperature variation, thermal expansion coefficient and bridge span length.

To consider the uncertainties associated with this load, super structure temperature and thermal expansion are considered as random variables as follows.

#### 5.4.1. Superstructure Temperature

The superstructure temperature is affected by the ambient air temperature, solar radiation, wind speed and direction, and so forth. The primary component of them is the ambient air temperature and can be assumed as the IAB's superstructure temperature [28]. Thus, based on Kim's research [9], for every 7 days, the IAB's superstructure temperature is a normal distribution with a mean value defined by (15) and standard deviation which have been tabulated in Table 4:

(15) $$\begin{array}{c} T_{\text{mean}}^{}\left(t\right)=\mu_{T}+A_{T}\sin\left(\omega t+\phi_{T}\right), \end{array}$$

where *μ*_*T*_ is annual mean temperature, *A*_*T*_ is annual mean temperature variation, *ω* is frequency (2*π*), *t* is analyzing time (year), and *ϕ*_*T*_ is phase lag. For three sites considered in this study the temperature statistics were established from the weather website [18] and summarized in Table 4.

#### 5.4.2. Thermal Expansion Coefficient

In this study, based on AASHTO LRFD [11] a nominal design value of 1.17 × 10^−6^/°C is considered for the thermal expansion coefficient of the IAB's deck steel girders. A bias of 1.0 and a COV of 0.1 using a normal distribution are used for this random variable [27].

## 6. Reliability Analysis of the Integral Abutment Bridge

In this section, the reliability analysis is performed for a basic IAB designed to satisfy the current AASHTO LRFD specifications for evaluating the safety level under seismic and thermal loads for its 75-year design life (a 75-year design life is used for an IAB [11]) for different limit states. As Piles moments and shears are most affected by the temperature change that accompanies an earthquake, the bending moment and shearing limit states of piled foundation are considered in this paper. For evaluating the safety level of a structure, first the reliability indexes are calculated for bridges under the effect of only earthquake load to evaluate the target reliability indexes [24]. Then these analyses are repeated for the same bridges subjected to the combination of seismic and thermal loads. Finally, resultant indexes will be compared with the target reliability indexes and the satisfaction of the target safety level for designed integral bridge according to AASTO LRFD specifications under the temperature and earthquake load combination will be investigated.

In this paper, the reliability indexes have been calculated by using Monte Carlo method. To combine the seismic and thermal loads, the scenario sampling method [22], and reliability analysis, the Rt Software has been used [23].

Geometric and structural properties of the basic IAB are described below.

### 6.1. Geometric and Structural Properties of the Basic IAB

**Figure 2:**
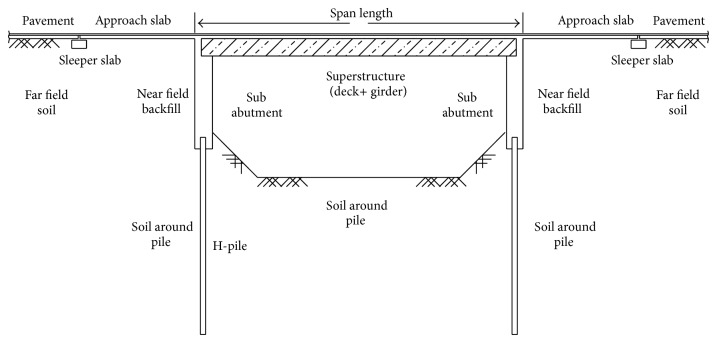
Longitudinal section of the IAB.

The considered IAB in this study is a one-span 40 m IAB having the longitudinal section as shown in Figure 2. The superstructure of the bridge is composed of concrete slab whit 20 cm thickness and steel beams at 2 m spacing. Each abutment of this bridge has 7 m height and 1 m wall thickness and supported on a single row of steel H piles with 12 m length at 1 m spacing. The section properties of deck girder and piles are given in Table 5.

The abutments backfill soil is assumed to be dense cohesionless soil with 30° angle of internal friction and a unit weight of 16.72 kN/m^3^.

**Figure 3:**
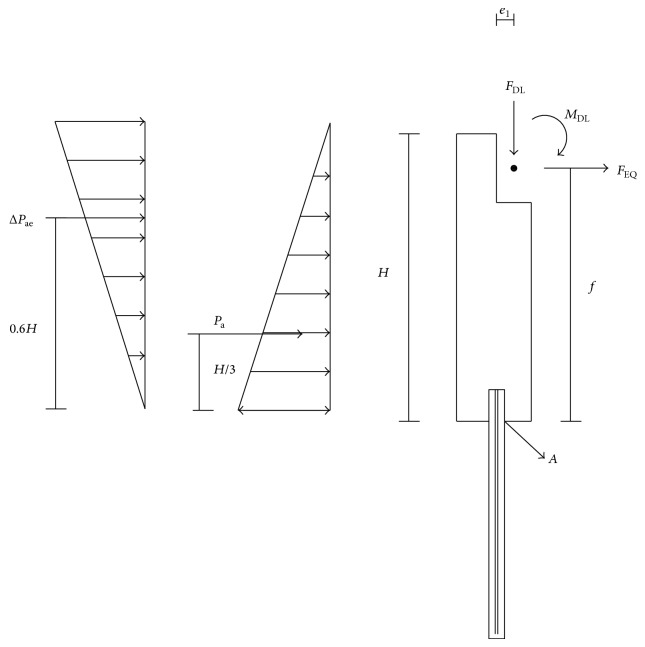
Free body diagram of IAB pile, dominant failure is bending at point *A*.

As described earlier, the piles moment and shear force are affected the most by the earthquake and thermal loads, thus, in this study bending moment and shearing failure limit state of the IAB piles at point *A* (where the pile connect to abutment) were considered for reliability analysis. The requirement moment and shear capacity were calculated to satisfy the current AASHTO LRFD specifications. The free body diagram of the basic IAB pile under applied load is shown in Figure 3, where *F*_DL_ = 198.16 kN is the permanent weight of superstructure, *M*_DL_ = 1.32 MN is the moment caused by the permanent weight of superstructure, *P*_*a*_ = 272.82 kN is the static active backfill force and acts on the H/3 from the bottom of abutment, Δ*P*_*ae*_ is the seismic active backfill force based on Mononobe and Matsue [29] and Okabe [30], and for San Francisco, Seattle, and Memphis it is equal to 819.33 kN, for New York it is equal to 166.28 kN, and for St. Paul it is equal to 17.27 kN. This load acts on the 0.6*H* from the bottom of abutment [31], *H* = 7 m is the abutment height, *e*_1_ = 0.25 m is the dead load eccentricity from the point *A*, and *F*_EQ_ is the equivalent earthquake force—described below—transferred from the IAB deck and acts on the distance *f* = 6 m from the bottom of abutment.

The equivalent internal earthquake force, *F*_EQ_, by using the nominal natural period of *T* = 0.41 s, the soil of type *D*, and the calculated spectra acceleration for 1000-year return period (7% probability of exceedance in 75 years) [11], is obtained as follows:

(16) $$\begin{array}{c} F_{\text{EQ}}=\frac{S_{a}\times W}{R_{m}}, \end{array}$$

where *S*_*a*_ is the spectral acceleration, *W* is the weight of structure, and *R*_*m*_ is the response modification factor. Based on AASHTO LRFD [11] by using a modification factor *R*_*m*_ = 1 the equivalent earthquake is equal to 307.54 kN for San Francisco site, 210.52 kN for Seattle, 176.38 kN for Memphis, 44.05 kN for New York, and 10.51 kN for St. Paul.

As the dominant AASHTO LRFD [11] load combination to design the considered pile is the extreme event I combination at point *A* (see Figure 3), the design equation used for calculating the nominal moment capacity is as follows:

(17) $$\begin{array}{c} \phi M_{\text{req}}=1.25M_{\text{DL}}+1.5M_{P_{a}}+M_{\Delta P_{ae}}+M_{\text{EQ}}, \end{array}$$

where *ϕ* is the resistance factor which for bending is equal to 0.9, *M*_DL_ = 1.37 MN m (= 0.198 × 0.25 + 1.32) is the total moment caused by permanent weight of superstructure, *M*_*P*_a__ = 0.64 MN m (= 0.273 MN × 2.333 m) is the moment caused by static active backfill force, and *M*_Δ*P*_*ae*__ is the moment caused by seismic active backfill force and is equal to 3.44 MN m (= 0.819 MN × 0.6 × 7 m) for San Francisco, Seattle, and Memphis, equal to 0.698 MN m for New York, and equal to 0.0725 MN m for St. Paul. *M*_EQ_ is the equivalent earthquake moment that is equal to 1.85 MN m for San Francisco, 1.26 MN m for Seattle, 1.058 MN m for Memphis, 0.264 MN m for New York, and 0.063 MN m for St. Paul.

Based on the extreme event I combination at point *A* (see Figure 3), the design equation used for calculating the nominal shear capacity is as follows:

(18) $$\begin{array}{c} \phi V_{\text{req}}=1.25V_{\text{DL}}+1.5V_{P_{a}}+V_{\Delta P_{ae}}+V_{\text{EQ}}, \end{array}$$

where *ϕ* is the resistance factor which for shearing is equal to 0.9, *V*_DL_ is the total shear caused by permanent weight of superstructure and usual 0, *V*_*P*_*a*__ = 0.272 MN is the shear caused by static active backfill force, and *V*_Δ*P*_*ae*__ is the shear caused by seismic active backfill force and is equal to 0.819 MN for San Francisco, Seattle, and Memphis, equal to 0.166 MN for New York, and equal to 0.0172 MN for St. Paul. *V*_EQ_ is the equivalent earthquake shear that is equal to 0.308 MN for San Francisco, 0.211 MN for Seattle, 0.176 MN for Memphis, 0.044 MN for New York, and 0.0105 MN for St. Paul.

Using (17) the requirement moment capacity, *M*_req_, is equal to 8.84 MN m for San Francisco, 8.18 MN m for Seattle, 7.96 MN m for Memphis, 4.024 MN m for New York, and 3.12 MN m for St. Paul. Using (18) the requirement shear capacity, *V*_req_, is equal to 1.706 MN for San Francisco, 1.6 MN for Seattle, 1.56 MN m for Memphis, 0.69 MN for New York, and 0.49 MN for St. Paul.

### 6.2. Reliability Analysis under Seismic Load

The reliability analysis of the IAB pile is performed using the models described in Section 4 and the free body diagram shown in Figure 3. Referring to Figure 3 the failure function for pile bending can be represented by following equation:

(19) ZM=MPile−(MDL+(ΔPae×0.6H+Pa×(H3))× λcyc+FEQ×f),

where *M*_Pile_ is the pile bending moment capacity, *M*_DL_ is the total moment caused by superstructure weight, *F*_EQ_ is the equivalent earthquake load transferred from superstructure defined by (14), *P*_*a*_ is the static active backfill force and acts on the *H*/3 from the bottom of abutment, Δ*P*_*ae*_ is the seismic active backfill force [29, 30] and acts on the 0.6*H* from the bottom of abutment [31], *H* = 7 m is the abutment height, *f* = 6 m is the distance earthquake load from point *A*, and *λ*_cyc_ is the model of the effect of cyclic loading on the pile.

Based on Figure 3, the failure equation for pile shearing can be represented as follows:

(20) $$\begin{array}{c} Z_{V}=V_{\text{Pile}}-\left(\left(\Delta P_{ae}+P_{a}\right)\times \lambda_{\text{cyc}}+F_{\text{EQ}}\right), \end{array}$$

where *V*_Pile_ is the pile shearing capacity.

**Figure 4:**
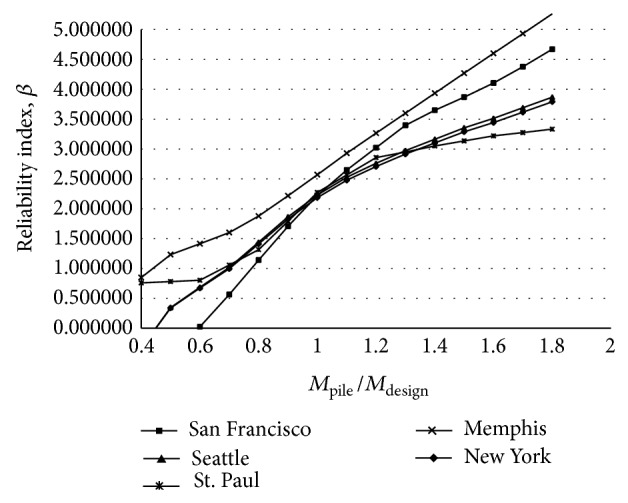
Reliability index for the bending of considered IAB's pile under earthquake load alone.

**Figure 5:**
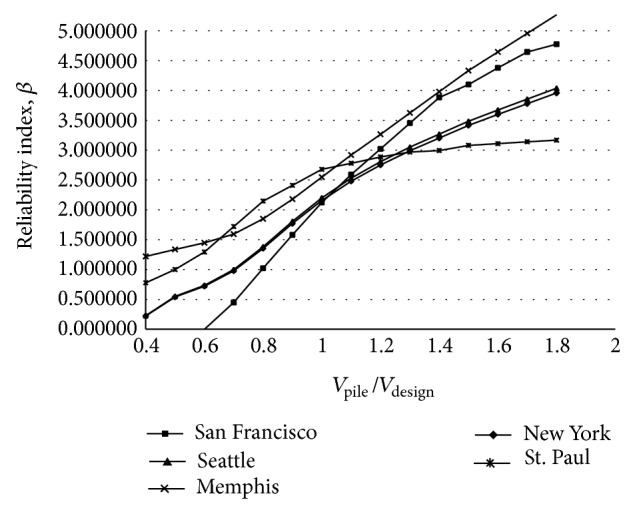
Reliability index for the shearing of considered IAB's pile under earthquake load alone.

Referring to (19)-(20), failure occurs when *Z*_*M*_ or *Z*_*V*_ are less than zero. All variables in (19)-(20) are considered random expect for abutment height, *H*, and distance earthquake load from point *A*, *f*. The statistical models used to describe the random variables are provided in Tables 1, 2, 3, and 4. In these failures limit state only earthquake load composed of time-depended and time-in depended random variables. The reliability analysis of the IAB pile was performed for five sites by using Monte Carlo simulation method using the Rt software [23]. Figures 4 and 5 show the reliability for the bending and shearing failure limit states for each of five sites as a function of pile moment and shear capacity, respectively. The abscissa of the plot is normalized such that a ratio of 1.0 indicates that the bridge is designed to exactly satisfy the AASHT LRFD [11] specifications requirements and reliability index corresponding to this ratio is used as the target reliability index, *β*_*T*_.  Figure 4 shows that the AASHTO LRFD [11] specifications using a nominal response modification factor *R*_*m*_ = 1 for pile bending limit state will produce a reliability index, *β*, between 2.12 and 2.57. The average from the five sites is equal to 2.28.  Figure 5 shows that for pile shearing limit state the average reliability index for five sites is 2.33 with a minimum index equaling 2.09 and a maximum value equaling 2.68.

### 6.3. Reliability Analysis for Combination of Earthquake and Uniform Thermal Loads

As it was written earlier, there is a temperature difference between temperature at any time of bridge life and bridge construction time temperature; hence, a uniform temperature load is applied to the bridge. Therefore, during earthquake the existence of a uniform temperature load is an obvious matter. Due to integrity structure of integral bridges, thermal and earthquake loads are important for these bridges and have determinant role in the performance of integral bridges.

Since most bridge design codes such as AASHTO LRFD have considered these two loads in combination, separately, in this study, designed integral bridge safety level evaluation according to AASHTO LRFD under thermal and earthquake loads combination was investigated. Also comparing the results with target safety level (which was the bridge safety level under seismic load alone) was studied. The research was done to understand whether the safety of these kinds of bridges (during structure life) under the combination of thermal and earthquake loads was satisfied or not?

#### 6.3.1. Combination of Earthquake and Thermal Loads (EQ + TU)

The IAB pile was analyzed to illustrate the combined effects of earthquake and thermal loads on the pile. The data from five earthquake sites described in Figure 1 were used. The uniform thermal load data were obtained from models developed by Kim [9] and weather website [18]. The reliability calculations of the IAB pile under considered combined loads follow the scenario sampling method described in Section 3.1. For this method the following assumptions are made.

- The reliability calculations are performed for the bending moment and shearing failure limit states of IAB pile.
- All earthquakes last 30 sec [24] and during this time it is assumed that the moment and shear at point *A* of the IAB pile remain at their highest value.
- All thermal loads last 7 days [9] and during this time the intensity of thermal load remains constant.
- The reliability analysis accounts for uncertainties which is associated with predicting the earthquake intensity, estimating the bridge pile response for given earthquake intensity, projecting the thermal load magnitude, and estimating pile moment and shear capacity.

Referring to Figure 3, the failure function of pile bending under combined load effects can be represented as follows:

(21) ZM=Mpile   −(MDL+(ΔPae×0.6H+Pa×(H3))     ×λcyc+FEQ+TU,75×f),

where *M*_EQ+TU,75_ = *F*_EQ+TU,75_ × *f* is the applied moment caused by the combined effects of the earthquake and uniform thermal loads in the period of *T* = 75 years.

Based on Figure 3, the failure equation for pile shearing can be represented as follows:

(22) $$\begin{array}{c} Z_{V}=V_{\text{Pile}}-\left(\left(\Delta P_{ae}+P_{a}\right)\times \lambda_{\text{cyc}}+F_{\text{EQ}+\text{TU},75}\right), \end{array}$$

where *F*_EQ+TU,75_ is the applied shear caused by the combined effects of the earthquake and uniform thermal loads. This combined effect is calculated by using scenario sampling [22, 23] method as described in Section 3.1.

The reliability analysis of the IAB pile was performed for five sites by using Monte Carlo simulation method, using scenario sampling method to combine seismic and thermal loads and using the Rt software [22, 23]. Tables 6 and 7 show the reliability index for the bending and shearing failure limit states for each of five sites. In these tables, the target reliability indexes are also shown and the resultant indexes under combined effect of seismic and thermal can be compared with them. As shown in Tables 6 and 7, for both limit states, for all considered sites when seismic and thermal loads combined, the reliability indexes and therefore the safety level have been reduced. Therefore, for pile bending and shearing limit states under combined effect of seismic and thermal loads, the AASHTO LRFD [11] specifications will not produce a responsible reliability index, *β*, by comparing resultant indexes with target indexes.

## 7. Conclusion

Integral abutment bridges (IABs) are jointless bridges that by eliminating the expansion joints have many advantages over conventional bridges. Due to the integrity of these bridges, among the loads acting on these bridges, seismic loads have major role in designing these bridges and readily transferred to substructure and affect the design of these components. As AASHTO LRFD like other developed bridge design codes consider temperature and earthquake loads separately in their specified load combinations for the design of bridges, according to the fact that there is a temperature difference between temperature at any time of bridge life and bridge construction time temperature, a uniform temperature load is applied to the bridge. Therefore, during earthquake the existing of a uniform temperature load is an obvious matter.

Then evaluating the safety level of IABs—designed by AASHTO LRFD bridge design code—under the combination of temperature and earthquake loads during the design life of IABs is important.

In this paper, by concerning the safety of the pile foundation of an IAB designed by AASHTO LRFD bridge design code under seismic load, the target safety level of IABs has been evaluated. Then by repeating these reliability analyses for the same bridge under combination of thermal and seismic loads and comparing calculated reliability indexes with target reliability indexes, it is shown that that for an IAB designed by AASHTO LRFD the reliability indexes have been reduced under combined effects and the target level of safety during its design life is not provided.

## Figures and Tables

**Table 1:** Dead load Statistics [13].

Load	Bias factor (*b*_*r*_)	Coefficient of variation (COV %)
Dead load (factory-made component)	1.03	8
Dead load (cast-in place component)	1.05	10
Asphalt wearing surface (88.9 mm assumed)	1	25

**Table 2:** Backfill Soil related statistics [14].

Variable	Bias	COV	Distribution type
Unit weight (*γ*)	1.0	7%	Normal
Fraction angle (ϕ_*s*_)	1.0	13%	Normal
Rankine coefficient (*K*_*P*_, *K*_*a*_)	1.5	20%	Normal
Cyclic effects, *λ*_cyc_	1.0	15%	Normal

**Table 3:** Earthquake load related statistics.

Variable		Bias	COV	Distribution type	Reference
Earthquake modeling factor, *λ*_eq_		1.0	20%	Normal	[15]

Spectrum modeling factor, *C*′	San Francisco	1.0	15%	Normal	[12]
	Seattle		25%		
	Memphis		25%		
	New York		30%		
	St. Paul		40%		

75-year PGA, *A*	San Francisco	from Figure 1	from Figure 1	from Figure 1	[16]
	Seattle				
	Memphis				
	New York				
	St. Paul				

Period modeling factor, *t*′		0.9	20%	Normal	[17]

Weight, *W*		1.05	5%	Normal	[15]

**Table 4:** Thermal load related statistics [18].

Variable	San Francisco	Seattle	Memphis	New York	St. Paul
Annual mean, *μ*_*T*_ °C	23.89	15.68	16.01	12.78	5.83
Annual variation, *A*_*T*_ °C	3.33	7	12.5	6.5	4.2
Daily standard deviation (°C)	6.59	5.68	6	5.75	6.5

**Table 5:** Steel sections properties.

Section	Size	Height (cm)	Flange width (cm)	Flange thickness (cm)	Web thickness (cm)
Girder	W 1000 × 975	111	43	9	5
Piles					
San Francisco	*H* 300 × 300 × 15 × 15	30	30	1.5	1.5
Seattle	*H* 300 × 300 × 12 × 12	30	30	1.2	1.2
Memphis	*H* 250 × 250 × 9 × 14	25	25	1.4	0.9
New York	*H* 200 × 200 × 12 × 12	20	20	1.2	1.2
St. Paul	*H* 200 × 200 × 8 × 12	20	20	1.2	0.8

**Table 6:** Reliability indexes for the bending moment limit state under combined seismic and thermal loads.

Site	Reliability indexes under combined effect, *β*_EQ+TU_	Target reliability indexes *β*_*T*_
San Francisco	2.21	2.223
Seattle	2.211	2.23
Memphis	2.52	2.57
New York	2.08	2.12
St. Paul	2.07	2.27

**Table 7:** Reliability indexes for the shearing limit state under combined seismic and thermal loads.

Site	Reliability indexes under combined effect, *β*_EQ+TU_	Target reliability indexes *β*_*T*_
San Francisco	2.10807	2.12296
Seattle	2.11144	2.20097
Memphis	2.28674	2.54758
New York	1.91	2.09
St. Paul	2.15	2.67815
